# PRIMASYS: a health policy and systems research approach for the assessment of country primary health care systems

**DOI:** 10.1186/s12961-021-00692-3

**Published:** 2021-03-06

**Authors:** Kabir Sheikh, Abdul Ghaffar

**Affiliations:** Alliance for Health Policy and Systems Research, World Health Organization, Geneva, Switzerland

**Keywords:** Primary health care, Health systems, Health systems assessment

## Abstract

For the renewed global impetus on primary health care (PHC) to translate into action at a country level, it will be important to strengthen existing ways of understanding and assessing country PHC systems. The architecture and performance of primary care systems are widely acknowledged to be crucial determinants of the health of populations in high-income countries as well as in low- and middle-income countries. There is no one-size-fits-all model of a country-level PHC system, and countries have implemented diverse models, adapted to and conditioned by their respective social, economic and political contexts. This paper applies advances in the field of health policy and systems research (HPSR) to propose an approach to the assessment of country PHC systems, using a compendium of 70 elements of enquiry requiring mixed quantitative and qualitative assessment. The approach and elements of enquiry were developed based on a review of policy and guidance documents and literature on PHC and HPSR and were finalized as part of a consultation of experts on PHC. Key features of the approach include sensitivity to context, flexibility in allowing for in-depth enquiry where necessary, systems thinking, a learning emphasis, and complementarity with existing frameworks and efforts. Implemented in 20 countries to date, the approach is anticipated to have further utility in a single country as well as in comparative assessments of PHC systems.

## Introduction

The architecture and performance of primary care systems are crucial determinants of the health of populations [1–3]. In 2018, countries around the world agreed to the Declaration of Astana, reaffirming their commitments to strengthen their primary health care (PHC) systems as an essential step towards achieving universal health coverage, and the United Nations Children’s Fund (UNICEF) and the World Health Organization (WHO) agreed to support countries to realize these commitments. If this renewed global impetus on PHC is to translate into action at a country level, it will be important to strengthen existing ways of understanding and assessing country PHC systems. However, there is no one-size-fits-all model of a country-level PHC system, and countries have implemented diverse models, adapted to and conditioned by their respective social, economic and political contexts. This paper applies advances in the field of health policy and systems research (HPSR) to propose an approach to the assessment of country PHC systems that acknowledges complexity and variability of context in analysing their structures, processes and outcomes. We start by reviewing the contested existing landscape of assessment frameworks and approaches that have been applied to PHC systems, followed by a detailed presentation of the Primary Health Care Systems (PRIMASYS) approach and elements of enquiry, and concluding with a discussion on its strengths and limitations.

## Framing and assessing PHC Systems

Frameworks and definitions of PHC reflect differing values and priorities and have shaped and driven global programmes and innovations at scale in many countries. Differences between frameworks have frequently engendered intense and long-standing debate. Typifying this is the debate between comprehensive and selective PHC.

The Alma-Ata Declaration, from the International Conference on PHC in 1978, heralded the emergence of PHC as a central concept in global health [4]. The declaration set forth a vision of PHC embedded in a comprehensive appreciation of the primacy of social justice and socioeconomic development in the improvement of health. Among the key tenets of this aspirational framework are (1) the invocation of health as a fundamental right, (2) a broad understanding of PHC as essential health care towards the production of “complete physical, mental and social wellbeing” among individuals and populations, (3) the inextricability of social, economic and political processes, particularly action to address social inequities, from the health of populations, (4) the preeminence of community leadership and ownership of planning and organization of PHC, and (5) the engagement of relevant non-health sectors in the production of better health [5].

The phase of worldwide attention on the aspirational principles of the Alma-Ata Declaration is described to have been rapidly followed by a radically distinct set of global policy reforms sometimes referred to as “selective PHC” [6]. Typified by UNICEF’s GOBI-FFF suite of targeted interventions, selective PHC involved prioritizing interventions by epidemiological importance and cost-effectiveness and was applied in poor country contexts where a few diseases were believed to be responsible for high rates of early-life mortality.

Variations of “comprehensive” and “selective” PHC approaches have respectively driven health care reforms in different low- and middle-income countries (LMICs), with widely varying results. The different starting points for the reform process, as well as different continuing circumstances in these countries, make direct comparability of improvements in health outcomes nearly impossible. However, some countries have enjoyed broad-based (Vietnam) or qualified successes (Sri Lanka), which have been attributed to combinations of external conditions (such as general socioeconomic development) or to specific systemic changes (such as district-level decentralization or innovative financing) [3]. A third term – diagonal approaches – has also been applied to describe the approach in countries (such as Egypt and Mexico) that are in planned transition between a phase of targeted interventions to more comprehensive systems reform [7].

Notable among subsequent frameworks that emerged are the work of Barbara Starfield and colleagues from the Johns Hopkins Populations Care Policy Center for the Underserved Populations, which focused on frontline service delivery and the first point of care, while retaining some features of original conceptions of comprehensive PHC [8]. Starfield’s work broke ground for the practical interpretation and implementability of broader normative principles in PHC, and for assessing PHC performance, through the introduction of the Primary Care Assessment Tool (PCAT)*.* The PCAT is based on a theoretical framework of primary care domains and characteristics and is one of the most widely implemented tools for PHC assessment to date [9].

The 1990s and early 2000s also witnessed the emergence of a distinct emphasis on health systems and the field of HPSR in the global health arena. A number of structured frameworks for health systems were developed and coincided with early attempts to couch PHC in the context of those frameworks. Dominant amongst a spectrum of early attempts to structure thinking on health systems were the WHO “building blocks” framework, and the “control knobs” framework emanating from the World Bank and partners. This move towards systems thinking provided early opportunities to explore and analyse structural determinants – such as health systems governance and resource generation – of the delivery of primary care services.

WHO’s Primary Care Evaluation Framework embeds the factors characterizing good PHC – access, comprehensiveness, coordination and continuity – in a broader context of health system building blocks, specifically governance and financing arrangements. WHO’s PCAT, consisting of a national questionnaire regarding organization and funding of PHC; a questionnaire for family doctors; and a questionnaire for service users, has been implemented in a range of settings [9]. The control knobs framework of health systems (namely financing, payment, organization, regulation, behaviour modification), initially developed by William Hsiao and further refined by Hsiao and colleagues, captures the manifold planning inputs that can help effect changes in the health sector and health markets [10]. One important advance in the control knobs framework is the PHC Performance Initiative (PHCPI) programme, which focuses on building high-performing PHC systems in LMICs. The PHCPI framework guides the application of discrete groups of indicators to assess the performance of country PHC systems, and its root causes [11–13]. PHCPI as well as the European Observatory’s European Primary Care Monitor [14] are primarily quantitative assessment approaches guided by a list of categorized and pre-identified indicators, enabling ease of comparability.

Recent frameworks of people-centred health systems (PCHS) also have significant relevance for PHC systems. The distinct new contribution of PCHS is in highlighting that health systems are social institutions, bound together by and contingent on relationships between diverse stakeholders operating in social and organizational contexts [15]. Recognition of the socially constructed, interdependent and self-organizing character of health systems adds a new dimension to understanding how PHC systems perform and how they can change – these have traditionally been seen through the lenses of command-and-control governance and economic rational choice approaches.

## A multidimensional framework of PHC systems

As can be gathered from the variety of definitions and frameworks, PHC systems have been the topic of widespread and intense debate. Views on framing PHC still often become polarized between (1) perspectives that inextricably link PHC to rights, social justice and to social, economic and political processes and determinants of health and (2) those that view PHC foremost as a level of care and emphasize its functions and measurable performance [16–18].

A systems lens suggests that the performance of a PHC system cannot be viewed merely in terms of its eventual outputs and outcomes (equitable access, responsive services and quality care), but must also be understood in terms of related and underlying structures and processes that explain these outcomes, and their interconnectivities [19]. There is ample evidence from high-income countries alike to indicate that structural elements of a PHC system (such as governance, financing, human resources and service organization) and key processes (such as policy implementation, regulation, supervision and the flow of information) are critical in shaping its outcomes [20], and somewhat more sporadic evidence from LMICs.

For example, good governance in non-health sectors were critical positive influences in almost all of the 14 countries identified with a comprehensive PHC system, in a review by Rohde and colleagues [3]. Lawn and colleagues reported that essential drugs policies have made an important contribution to PHC [6]. On the other hand, Lawn and colleagues have observed that community participation, intersectoral engagement and the appropriate use of technology are among the weakest-performing elements in PHC performance globally [6]. Other key problems include ever-increasing task lists for overburdened primary health care workers that necessitate long-term human resource planning, better training and supportive supervision.

Integration of community- and facility-based care, comprehensive and selective systems [6], a nationally agreed package of prioritized and phased PHC that all stakeholders are committed to implementing, attention to district management systems, and consistent investment in primary health care extension workers linked to the health system [3] have been observed to be important factors contributing to the effectiveness of PHC systems. Equally important are political and financial commitment to PHC, and the efficient use of data to direct priorities and assess progress, especially at a district level.

Kringos and colleagues propounded an approach to the assessment of primary care systems in Europe, which outlines the multidimensional framing of PHC systems in terms of structures, processes and outcomes [20]. Here, “structures” refer to the relatively unchanging elements of primary systems – institutional, infrastructural and economic – that shape and condition the delivery of effective services. Structural elements are broadly classified into governance, financing, human resources and service organization. “Processes” refer to the dynamic phenomena and events that occur in planning, regulating, implementing and monitoring primary care systems, and influence their ultimate performance. Health systems “Outcomes”, as distinct from “health outcomes”, are manifestations of the performance of PHC systems at the frontlines. Key outcome categories include equitable access to primary care services at scale, the appropriateness and responsiveness of those services to people’s needs, and the quality and safety of the services that people ultimately receive.

**Fig. 1:**
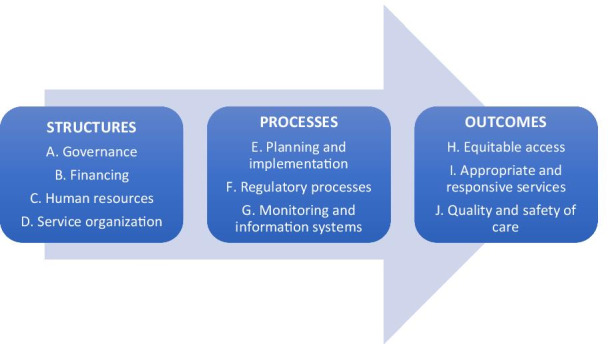
Thematic framework for PHC systems (adapted from Kringos et al. [19])

Under the themes in the framework, a total of 70 elements of enquiry are identified and enlisted as reflecting the performance of a country PHC system (see Table 1 for the full list). These elements were developed based on a review of policy and guidance documents and published literature on PHC and HPSR. The elements were organized into a framework adapted from Kringos et al. (Fig. 1) and finalized as part of a consultation of PHC experts hosted by the Alliance for Health Policy and Systems Research (www.who.int/alliance-hpsr/projects/PRIMASYS_Expert_Consultation_Final_Report.pdf?ua=1). The experts at the consultation also drew on the criteria listed in Table 2 to divide the elements of enquiry into “fixed” and “flexible” elements. “Fixed” elements are to be assessed in the same manner in each setting and are deemed to be comparable across different country settings. “Flexible” elements are specific to the country in question. (Table 2 outlines criteria for the identification of fixed elements.)

**Table 1:** Elements of enquiry (fixed elements in bold text)

**Structures** refer to the relatively unchanging elements of primary systems – institutional, infrastructural and economic – that shape and condition the delivery of effective services. Structural elements are broadly classified into governance, financing, human resources and service organization. A. Key aspects of health system governance, as a high-level function, that can be recognized as being immediately relevant to the performance of PHC systems include: **1. Existence of a national policy statement on health equity, universal health coverage and/or health rights** **2. Presence of institutional mechanisms to represent citizen voice and civil society engagement in health service organization and planning** **3. Relative role of government, private for-profit and not-for profit sectors, and development partners in the delivery of health services** 4. Extent of de jure decentralization of decisions for health care management and services 5. Presence of institutional and legal mechanisms for feedback and action on user grievances in the health sector 6. Presence of institutional mechanisms to engage other sectors (water, agriculture, education, transportation, etc.) for action on social and environmental determinants of health 7. Existence of systems to identify, measure and respond to disease burden in the population, including emerging health priorities such as multiple morbidity, mental health, and epidemics B. Arrangements and systems for financing health, and improving financial flows in the health sector that are relevant for PHC systems are enlisted below: **1. Overall government commitment to tax-funded health care, reflected as total government allocation for health as a proportion of the GDP** ** 2. Government’s relative commitment for primary-level care, reflected as a proportion for PHC out of total government allocation for health** **3. Extent and magnitude of inequitable and inefficient financing mechanisms, reflected in such measures as proportion of out-of-pocket expenditure, proportion of households experiencing catastrophic health expenditure, prevalence of user fees** **4. Extent (depth) of financial coverage of services and conditions at point of care, that is, selective or comprehensive coverage** 5. Extent and depth of de jure financial decentralization, presence of schemes for financial decentralization 6. Extent of illegitimate fund outflow due to corruption (where information is available) in the health system 7. Types and extent of purchasing arrangements in force, including contracting, franchising, social insurance and pay-for-performance 8. Time trends of increases in health care costs and household expenditure, reflecting growing burden on households 9. Government and development partner commitment to strengthening horizontal systems, as reflected in the ratio of allocation for general health services with respect to vertical health programmes C. Aspects of the availability of human resources for health and health worker education and support systems that directly influence the performance of PHC systems include: **1. Proportions of doctors/nurses/midwives/paramedical workers/community health workers (CHWs) engaged in providing PHC, and relative geographical distribution of each group** 2. Relative distribution of PHC providers in public vs private employment 3. Proportion of informal and untrained providers out of the total PHC workforce, and schemes to engage them 4. Proportion of practitioners of traditional, complementary and alternative (TCA) systems of medicine out of the total PHC workforce, and schemes to engage them 5. Adequacy, quality and appropriateness of professional education and in-service training for different cadres of PHC professionals 6. Trends in greater professionalization of PHC, such as medical specialization in primary or family care, nurse practitioner programmes, and career advancement for CHWs D. Service organization refers to the organizational arrangements that can facilitate the efficient, equitable and appropriate delivery of integrated, high-quality PHC services: **1. Presence of systems for referral and counter-referral between different tiers of care, including gatekeeping, patient transport and information tracking** ** 2. Policies to deploy PHC teams with clearly identified roles (rather than stand-alone frontline providers) and responsibility for a specified population** ** 3. Existence of CHW programmes, and scale achieved** **4. Extent of de jure integration of vertical programme structures reflected in a common chain of command, fund flow and infrastructure** 5. Extent of de jure integration of PHC with public health functions reflected in a common chain of command, fund flow and infrastructure 6. Extent of de jure diversification and substitution of PHC providers 7. Clearly demarcated strategies for in-service support, including decision support, training, recognition and retention for frontline providers 8. Extent of use of mobile phone and rapid diagnostic technologies for decision support, treatment support, or advice and counselling **Processes** refer to the dynamic phenomena and events that occur in planning, regulating, implementing and monitoring PHC systems, and influence their ultimate performance. E. It is widely recognized that in the health systems of many LMICs, de facto conditions frequently do not follow de jure governance arrangements. Wherever possible, it is important to ascertain what actually happens (rather than what is expected to happen) in the planning and implementation of PHC services **1. Effectiveness of systems to identify, measure and respond to disease burden in the population including emerging health priorities such as multiple morbidity, mental health, and epidemics** 2. Prevalence and effectiveness of institutional mechanisms to ensure financial tracking and accountability and counter corruption in the health sector **3. Effectiveness of institutional mechanisms to represent citizen voice and civil society engagement in health service organization and planning** **4. Effectiveness of institutional mechanisms to engage other sectors (water, agriculture, education, transportation, etc.) for action on social and environmental determinants of health** 5. Status and successes (if evaluated) of prevailing purchasing arrangements, including provider contracting, franchising, social insurance and pay-for-performance 6. Capacity and environment for decentralization of health system functions and financing decisions at different levels, and character of federal-state-district relationships in the health care sector 7. Extent of de facto integration of vertical programme structures reflected in a common chain of command, fund flow and infrastructure 8. Extent of de facto integration of PHC with public health functions reflected in a common chain of command, fund flow and infrastructure 9. Extent of and successes (if evaluated) in diversification and substitution of PHC providers 10. Effectiveness of integration and utilization of TCA and informal medical providers in delivery of PHC 11. Effectiveness of systems for referral and counter-referral between different tiers of care, including triage, patient transport and information tracking 12. Extent of actual presence of PHC teams with clearly identified roles (rather than stand-alone frontline providers) and responsibility for a specified population 13. Status and successes (if evaluated) of use of mobile phone and rapid diagnostic technologies for decision support, treatment support, or advice and counselling	

F. Regulatory processes reflect the government’s ability to ensure the conditions for fair competition and high quality in markets for PHC. While critically important to curb distortions associated with market failures in mixed health systems, such knowledge is typically not easy to objectively ascertain in a short span of time, and is better understood through key informants, and by tracking existing research. 1. Government capacity for managing partnership agreements with non-state sector service providers 2. Government capacity to regulate conflicts of interest, and enact anti-trust laws and fair competition norms in the health care sector ** 3. Government capacity to regulate quality of services and medical products in the non-state health care sector, supported with provisions for punitive action** **4. Government capacity to regulate standards of professional education for PHC providers** 5. Effectiveness of institutional and legal mechanisms for feedback and action on user grievances in the health sector G. Monitoring and information systems are crucial factors in ensuring internal accountability and the alignment of publicly delivered PHC services with their intended functions. 1. Health worker accountability to communities, as reflected in the prevalence of health worker absenteeism ** 2. Regular supervision, performance review of facilities and PHC teams** **3. Existence and reliability of health information management systems for tracking major service delivery indicators and health outcomes** 4. Existence of systems for utilization of anonymous health management information system (HMIS) data to decision-makers at different levels for planning purposes 5. Existence of institutional mechanisms for supportive supervision of CHWs Health systems** outcomes**, as distinct from “health outcomes” (beyond the scope of these health systems assessments), are manifestations of the performance of PHC systems at the frontlines. Key outcome categories include equitable access to PHC services at scale, the appropriateness and responsiveness of those services to people’s needs, and the quality and safety of the services that people ultimately receive. H. The first and most apparent outcome of a successful country PHC system is equitable access at scale of PHC services. ** 1. Geographic availability and equity: the extent and equitable presence of functioning PHC facilities, across rural, peri-urban and urban locations** ** 2. Socioeconomic equity: the equitable presence of functioning PHC facilities, across locations defined by social (such as sectarian) and economic differences within a country** ** 3. Extent of acceptance and equitable utilization of functioning PHC services, reflected in the proportion of essential services utilized (such as institutional deliveries, immunization coverage and tuberculosis (TB) treatment completion), in absolute terms, and disaggregated across social and economic categories (where information is available)** 4. Extent of acceptance and equitable utilization of functioning PHC services, reflected in documented social and/or economic barriers to accessing functioning PHC services, in the absence of geographical barriers I. Appropriate and responsive care: care services must be organized in a manner that is responsive to the long-term needs of users, and reflect the role of PHC services as social and community-embedded institutions 1. Health coproduction: systems to ensure users’ views are respected and accounted for in arriving at therapeutic decisions 2. Relational continuity: existence of provisions for user engagement with a single point of contact from the PHC team, over a period of time 3. Longitudinal continuity: existence of provisions for user engagement with PHC teams across separate illness episodes 4. Informational continuity: existence of provisions for organized collection of user’s medical information available to the PHC team **5. Comprehensiveness of services: availability of combination of promotive, preventive and curative services at point of care** **6. Therapeutic comprehensiveness: availability of care and/or appropriate referral for a wide range of complaints and conditions at point of care** 7. Regular availability and utilization of necessary equipment and medical products for treatment of those complaints and conditions at point of care 8. Productive efficiency: steps taken to minimize expenditure and opportunity cost for users in accessing care, without compromising outcomes 9. Efficiency of workers: steps taken to assess and streamline frontline worker caseload, duration of consultations and prescribing practices reflect efficiency, without compromising outcomes 10. Technical efficiency: steps taken to streamline and reduce utilization of resources and costs of providing care, without compromising outcomes J. Quality and safety of care is of paramount importance and finds reflection equally in the perceptions of users, and in adherence to the technical parameters that guide standard care practices. **1. User satisfaction with PHC services, for example as reflected in user perception surveys** **2. Frontline provider adherence to standard treatment practices, as reflected in monitoring data or survey results on treatment of major therapeutic categories such as chronic diseases, mental health, maternal & child health, infectious diseases** **3. Existence of arrangements to regularly assess and monitor patient and health worker safety in PHC facilities**

**Table 2:** Criteria for fixed elements of enquiry

*Importance*: consensus on the importance of the element, as a fundamental and universal reflection of performance of PHC systems *Breadth*: sensitive enough to be relevant for more than one, or even several, elements of PHC systems *Uniform interpretation*: consensus on global interpretation and approach to assessment or measurement, including similar types of data sources used in different settings *Reliability and availability of information*: more likely to be assessed using reliable and widely available sources of information across different country settings *Part of existing tool*: wherever possible, already reflected in existing PHC assessment tools	

## The HPSR approach

Recent thinking around the practice of HPSR and the roles of HPSR researchers, particularly in LMICs, indicates the need to extend the researcher’s role to go beyond data generation and analysis, to encompass a fuller range of opportunities and potential responsibilities [21, 22]. In keeping with these advances, an approach to the assessment of country PHC systems was developed that goes beyond simple data collection and analysis and extends to engaging varied stakeholders with a view to situating and contextualizing the research and its outputs and promoting learning and reflection at multiple levels. The core features of the methodological approach are outlined in Table 3, and the specific aims of the approach (PRIMASYS) are as follows:

- To summarize key aspects of the structures, processes and outcomes of the country’s PHC systems that reflect their performance
- To elaborate specific “pathways” that have contributed to notable successes and/or failures in the country’s PHC systems
- To promote learning among relevant stakeholders to motivate policy change

**Table 3:** Core features of the PRIMASYS approach

*Context sensitivity*: provides guidance for context-sensitive and context-specific enquiry through flexible elements of enquiry, and engagement of country-level key informants *Flexibility*: allows for the deeper description of context-specific pathways of reform and explanations of failures, through micro-case studies utilizing qualitative methods *Systems thinking*: promoting the conception of PHC systems as a composite of variably interlinked elements, including structural elements (governance, financing, service organization) *Learning emphasis*: integrates different forms of learning for policy *Complementarity:* builds on current advances and efforts	

**Fig. 2:**
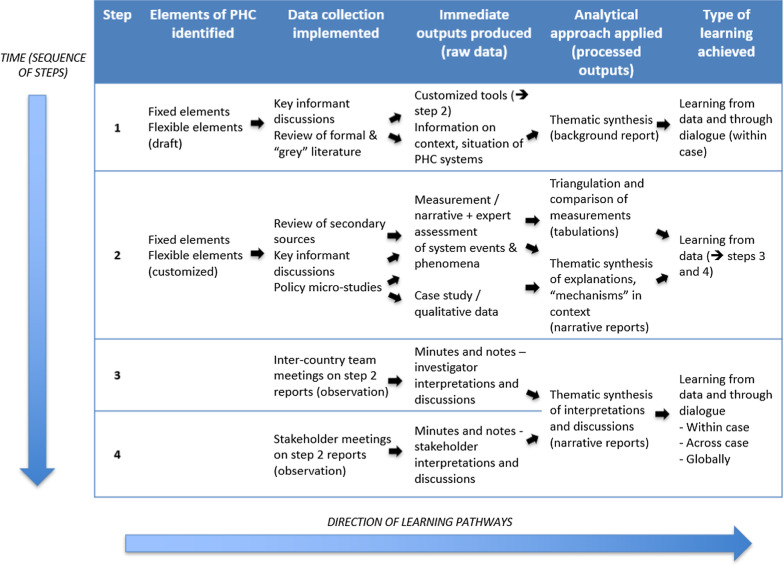
Steps for country PHC system assessments

The four practical steps of the PRIMASYS approach are described below and summarized in Fig. 2. Step 1 crucially focuses on customization of the methodology and is premised on the importance of contextualizing the enquiry by accessing experiential knowledge of stakeholders and unpublished and grey literature, which is not always immediately accessible to external actors [22, 23]. This step is crucial in building the credibility of the approach and ultimately facilitating joint learning – by initiating engagements with key informants and stakeholders around the objectives and methods. The main components of this step are as follows:

- *Preliminary assessment*: The assessment team undertakes collation and commencement of review of relevant peer-reviewed and grey literature, and discussions with selected key informants, to understand the context and current situation of PHC systems in the relevant country. Relevant information on the national demographic and epidemiological profile, and an overview of social, political, economic and environmental characteristics and determinants of health, with a focus on equity, will be obtained.
- *Selecting the “flexible” elements of enquiry* [24]: This step involves finalization of a customized list of indicators, tracer themes, and themes for micro-studies. Reviewing relevant literature and discussions with in-country key informants and stakeholders around the applicability and significance of different elements of the framework helps to identify the flexible elements of enquiry. Key informants are also asked to identify key PHC policy successes or failures as topics for the policy micro-studies.
  Step 2 is the main data collection step of the country health systems assessments – methods consist of collation and tabulation of available secondary data, policy “micro” studies to investigate specific pathways of change, and key informant interviews to contextualize and triangulate the emerging data.
- *Indicators and tracer themes* (see Table 1): A key task of the assessment is to access relevant and reliable secondary sources of information to measure indicators and obtain information on the identified tracer themes. Sources of relevant information include existing routine national health data, health facility records, global- and country-level health databases, large-scale population-based surveys and facility assessments, and published and unpublished articles and study reports. Indicator data may be gathered from multiple sources, if available, and triangulated across sources to avoid major inconsistencies and errors.
- *Policy micro-studies* are case studies addressing a focused research question or theme of importance in the country context, and employing a recognized HPSR approach, such as policy analysis or theory-driven evaluation [25, 26]. Methods are typically qualitative, including in-depth interviews with health system actors and key informants, and review of policy documents, followed by thematic analysis, and supported by review of the relevant literature. Micro-studies help by capturing pathways of change and complex interconnectivities between system elements and providing insights into the scenario of PHC systems in the country at large. In addition to documenting pathways of success, attention is also given to relevant negative cases, such as barriers to reforms or notable failures of PHC systems.

Step 3 consists of sharing between country teams of their respective findings, to enable comparisons and interpretation of emerging themes. When it comes to findings that are not directly comparable or generalizable, cross-case analysis becomes a dynamic process that is contingent on the insight and creativity of the individuals who are involved in engaging collectively with the findings and ideas. Case study and qualitative research embraces principles of theoretical or analytical generalization or comparison, rather than direct comparison. To quote Gilson, comparison is “grounded in a process of abstracting from the specifics of one case to ideas that encompass several cases”. It is precisely the delineations of pathways or “mechanisms operating in context” that throw up explanations that are rich in their specificity, but also sufficiently general to find resonance in different contexts [23].

Step 4 consists of meetings with in-country stakeholders to elicit their interpretations of the findings of the health systems assessments and engage them on their utility and application for PHC systems and policy. Progress in analysis and learning in such cases occurs through the process of dialogue, rather than through the solitary perusal of data [27].

## Discussion

According to Bennett and Peters [28], national health systems assessments (HSA) should meet the following criteria:

- Relevance, addressing the purpose for which it was designed
- Trustworthiness in terms of being of high quality, rigorous and credible in the eyes of stakeholders
- Coherence, considering the health system as a meaningful whole with linkages across system components

Relevance implies the fidelity of the assessment to its ultimate purpose and intention. The rationale of this health system assessment is to generate accurate and useful knowledge and information about country PHC systems, to facilitate learning for diverse stakeholders at subnational, national and global levels, in turn motivating informed and harmonized policy improvements and reforms. The approach was developed to add value on such aspects that are still poorly understood, and complement and synergize, rather than replace or compete with advances made through other existing tools and approaches. Within the context of the high-level framework (Fig. 1), this approach enabled considerable country-specific customization of elements of enquiry: the mixed methods in data collection, and an approach to analysis and interpretation within and across differing contexts that embraced complex causality and focused on promoting learning for useful change through dialogue, rather than only on mechanistic comparisons [23].

Twenty country case studies were undertaken by country-based research teams supported by the Alliance for Health Policy and Systems Research, through a financial grant from the Bill and Melinda Gates Foundation. In each instance the case studies addressed key knowledge gaps in the pathways and mechanisms through which country PHC systems (or in the case of large countries with decentralized authority: state and province) have achieved successes or met with challenges. These findings are detailed in a comparative analysis of key findings from the case studies by Langlois and colleagues [29]. Detailed reports of these country case studies are available here: https://www.who.int/alliance-hpsr/projects/primasys/en/

Trustworthiness implies quality, rigour and credibility in the eyes of stakeholders, which is a critical factor in ensuring buy-in and facilitating learning from the process. Quality and rigour are partly reflected in the quality and reliability of the secondary data that are available for national health systems assessments of this nature. Frequently, judicious decisions, involving deliberations and triangulation with country key informants, were made around how (and whether) to use and interpret different data sets. At the same time, all primary data collection contributing to the assessment, including surveys and in-depth case studies, observed standard norms of rigour and quality that apply for quantitative and qualitative health systems research [14]. Likewise, standard research ethics norms were observed, and relevant in-country ethics permissions were obtained. Reviewing relevant peer-reviewed and grey literature and engaging in-country key informants and stakeholders continuously – before, during and after the assessment – facilitated credibility [30].

Finally, the criterion of coherence requires that the health system be regarded as a meaningful whole with linkages across system components. The framework consistently underlines the interlinkages between these different elements as they contribute towards the successes and failures of PHC systems [19, 31, 32].

The proposed approach also has some potential limitations. The main among these is the length of elements of enquiry, some of which require detailed and nuanced exploration. The demarcation of fixed and flexible elements provides an opportunity for investigators to address this issue by prioritizing flexible elements that have more relevance for the specific country context. The mixed quantitative and qualitative approach proposed also challenges direct comparability across settings. However, this level of granularity and depth of analysis is important to be meaningful to local stakeholders and stimulate learning, even as it allows comparability of the more narrowly defined elements across contexts.

It is well established that there is no one-size-fits-all model of a country-level PHC system, and countries have implemented diverse models, adapted to and conditioned by their respective social, economic and political contexts [3, 9, 33]. Hence, while higher-level explanations are feasible, the specific pathways or “mechanisms” of how PHC systems perform in context need to be identified through context-sensitive, empirical enquiry [2]. The experiences of implementing this approach in 20 LMICs shows that it has utility in enabling the assessment of country PHC systems in a way that acknowledges complexity and variability of context. We hope that the approach will find even wider utility in country-focused as well as comparative assessments of PHC systems.

## Data Availability

Not applicable since this is a commentary.
